# Beliefs about the Nature of Forgiveness and Avoidance of an Offender among Chinese College Students

**DOI:** 10.3390/bs13090747

**Published:** 2023-09-07

**Authors:** Zhaoyue Yi, Di Wu, Mianlin Deng

**Affiliations:** Department of Psychology, Shanghai Normal University, Shanghai 200234, China

**Keywords:** belief about the nature of forgiveness, unconditional forgiveness, conditional forgiveness, forgiveness, explanation, behavioral avoidance, interpersonal offense

## Abstract

Previous research on beliefs about the nature of forgiveness (unconditional and conditional) has focused on their effects on health and well-being. However, little is known about how they influence victims’ responses to interpersonal offenses. Given that avoidance is a common response to offenses during early adulthood, this study investigated the relationships between beliefs in unconditional and conditional forgiveness and avoidance of an offender among Chinese college students, the mediating role of forgiveness, and the moderating role of whether or not the offender explains the offense. Participants were 423 Chinese college students. They were asked to recall an unforgettable incident in which another person had offended them, and then completed the following measures: the offender’s explanation, the belief in unconditional/conditional forgiveness, forgiveness, and avoidance of the offender. The study found that: (1) Victims’ belief in unconditional forgiveness negatively predicts their avoidance of an offender, whereas their belief in conditional forgiveness positively predicts the avoidance of an offender. (2) Forgiveness mediates the relationships between beliefs in unconditional and conditional forgiveness and avoidance of an offender. (3) The offender’s explanation moderates the relationships between the belief in conditional forgiveness and forgiveness, as well as avoidance of an offender.

## 1. Introduction

An interpersonal offense is a type of interpersonal stressor in which individuals perceive morally incorrect treatment from others and experience pain [1]. They typically arise from conflicts in values, rules, or interests among people [2]. Various forms of interpersonal offenses exist, including deception, emotional abuse, insults, and physical assault [2,3]. These offenses have adverse effects on victims’ emotional, physical, and social health [2,4,5].

Avoidance of the offender is a common behavioral response to interpersonal offenses [6]. This can protect the victim from further harm and also serve as covert revenge against the offender, as aggressive and overt forms of revenge are generally discouraged in society and may trigger retaliation from the offender [7]. However, avoidance negatively affects interpersonal relationships and mental health. For example, the avoidance of wrongdoers hinders conflict resolution and relationship restoration [8,9,10]. In addition, avoidance may lead to elevated anxiety, emotional exhaustion, and major depression because it prevents the victim from actively resolving and actually letting go of the conflict [11,12,13]. Therefore, exploring the factors that mitigate victims’ tendency to avoid transgressors can improve their interpersonal relationships and psychological well-being.

Forgiveness, defined as an intrapersonal process involving a prosocial change toward an offender (e.g., relinquishing anger and resentment) [14,15,16], plays an important role in determining an individual’s behavioral response (e.g., avoidance or seeking reconciliation) to an offender [15,17,18]. Previous research has shown that victims’ personal characteristics, such as empathy, personality, and religiousness, can promote forgiveness and reduce their avoidance of offenders [19,20].

Consistent with the aforementioned research, a recent study has revealed that personal beliefs about the unconditional and conditional nature of forgiveness can also influence forgiveness of an offense and overall well-being [21]. The belief in unconditional forgiveness posits that a victim can forgive independently of the offender’s behavior. Conversely, the belief in conditional forgiveness proposes that the offender must meet certain conditions (e.g., showing repentance) before a victim can forgive [21,22]. However, beliefs regarding the nature of forgiveness have not been sufficiently studied [22]. Whether beliefs in unconditional and conditional forgiveness have distinct effects on individuals’ avoidance of an offender, and if so, how and when these effects occur remains unknown.

Furthermore, existing studies on beliefs in unconditional and conditional forgiveness have primarily focused on individualistic Western cultures [21,22,23,24]. Whether these beliefs have the same influence on collectivistic non-Western cultures remains unclear. In individualistic cultures (e.g., America), victims’ personal goals (e.g., personal healing and inner peace) drive their responses to interpersonal offenses [25]. These responses are considered personal decisions and can be influenced by victims’ personal characteristics [26,27]. By contrast, collectivistic cultures (e.g., China) prioritize relational interdependence and social harmony. Cultural expectations of maintaining social harmony may influence how victims respond to offenses [25]. Therefore, responses to offenses in collectivistic countries are not solely personal decisions, as they are in individualistic countries [26,27]. It is crucial to examine whether personal beliefs in unconditional and conditional forgiveness influence victims’ responses to offenses (e.g., forgiveness and avoidance of an offender) in collectivistic cultures.

To address these research gaps, this study investigated the effects of the two beliefs about the nature of forgiveness on avoidance of an offender in collectivistic cultures. Moreover, this study examined the mediating role of forgiveness and the moderating role of offender’s explanation in the relationships between the two beliefs of forgiveness and avoidance. These examinations contribute to our understanding of how and when beliefs in unconditional and conditional forgiveness influence avoidance of an offender. Additionally, considering that interpersonal offenses and conflicts are prevalent in college life [28,29], and avoidance is a common behavioral response to offenses during early adulthood [6,30], this research employed Chinese college students as participants to explore the research questions.

The remainder of this paper is organized as follows: Section 2 provides a literature review focusing on the relationship between beliefs in unconditional and conditional forgiveness and avoidance of an offender, the mediating role of forgiveness, and the moderating role of whether the offender provides an explanation. In this section, we progressively propose three clusters of hypotheses and a moderated mediation model. Section 3 reports the method used to test the hypotheses and the moderated mediation model. Section 4 provides a detailed report of the results. Section 5 and Section 6 present a general discussion of the findings and conclusions, respectively.

## 2. Literature Review and Hypothesis Development

### 2.1. The Relationship between Beliefs about the Nature of Forgiveness and Avoidance of an Offender

Victims of interpersonal offenses have different beliefs about the nature of forgiveness [21,22]. The belief that forgiveness is unconditional is based on the idea that individuals can forgive an offense and respond to the offender on a moral principle of beneficence [31]. This principle encompasses virtues such as compassion, unconditional worth, generosity, and moral love toward the offender. According to this belief, forgiveness is seen as a voluntary choice made by victims and can therefore be unconditional, regardless of the actions of the offender. In this sense, forgiveness is a unilateral process in which a victim can forgive an offense without requiring the active participation of the offender [22,32]. In contrast, the belief in conditional forgiveness regards forgiveness as a social process in which a victim can grant forgiveness only if the wrongdoer takes certain actions or meets specific conditions, such as showing remorse, apologizing, or repairing [22]. That is, forgiveness is negotiated and granted through the principle of justice [32].

Previous studies have compared the effects of beliefs in unconditional and conditional forgiveness on well-being and relational outcomes. They found that belief in unconditional forgiveness enhances well-being [23,24], relational satisfaction, trust, and cooperation [33,34]. In contrast, belief in conditional forgiveness triggers psychological stress and a mortality risk, as well as a low level of well-being among victims of interpersonal offenses [23,24]. This inhibits the development of positive attitudes toward the offender and reduces relational closeness and satisfaction [33,35].

Possible explanations for the distinct effects of beliefs in unconditional and conditional forgiveness on mental health and interpersonal relationships are as follows: If a victim believes that forgiveness is unconditional and an act that they can exclusively control, independently of the offender’s behavior or attitude, it enables the victim to free themselves from the shackles of enmity and resentment binding them to the offender [22]. This may increase the victim’s sense of agency—the capacity to initiate actions autonomously and control one’s own life situation [2,36], which is related to good mental health, high relationship quality, and pro-relationship behavior [37,38,39]. In contrast, if a victim believes that forgiveness should be conditional on the offender acknowledging, apologizing, or compensating for their wrongdoing, this will reduce the victim’s sense of agency over the situation and shift more power to the offender [22]. Previous studies have indicated that a lack of power or agency activates behavioral inhibition tendencies and social vigilance [40,41,42,43]. This intensifies the victim’s social defensiveness elicited by the interpersonal offense.

Based on the explanations presented above, we argue that belief in the unconditional nature of forgiveness may alleviate a victim’s social defensiveness, and thus promote positive, cooperative responses to an interpersonal offense. In contrast, belief in the conditional nature of forgiveness may trigger a high level of social vigilance, which may lead the victim to display more defensive interpersonal reactions, such as avoidance of the offender. Therefore, we assume the following:

**Hypothesis** **1a.** *A victim’s belief in unconditional forgiveness negatively predicts their avoidance of an offender*.

**Hypothesis** **1b.** *A victim’s belief in conditional forgiveness positively predicts their avoidance of an offender*.

### 2.2. The Mediating Effect of Forgiveness

Forgiveness is a complex response to interpersonal offenses and has been conceptualized as involving prosocial changes in emotions, behavioral intentions, and behaviors [1,9,44]. However, scholars have argued that forgiveness is an intrapersonal experience that should be separated from external behaviors, such as avoidance or reconciliation [15,16]. For example, victims may attempt to reconcile with an offender in the absence of forgiveness if they need to continue studying or working together. Therefore, the conceptualization of forgiveness in this study emphasizes emotional change as the key aspect of forgiveness, rather than focusing on behavioral intentions or behaviors. Specifically, based on the emotion regulation theory [15], we define forgiveness as an emotion-regulation process during which the victim of an interpersonal offense improves their emotions toward the offender, including decreasing negative emotions (e.g., resentment and sadness) and/or increasing positive emotions (e.g., compassion and sympathy) toward the offender [15]. At its core, forgiveness represents an intrapersonal process of emotional change that involves setting emotional goals (e.g., letting go of negative emotions toward the offender) and regulating the current emotional state (e.g., feeling angry at the offender) to reach the goals over time [15]. There are various emotion regulation strategies, such as acceptance, distraction, and cognitive reappraisal, that aggrieved victims can use to enhance their emotions [45]. These strategies can be intrapersonal or interpersonal [15]. For instance, an intrapersonal strategy involves a victim cognitively reappraising an offense (e.g., the offense may not have been intentional), while an interpersonal strategy involves seeking an apology or explanation from the offender.

Beliefs about the unconditional and conditional nature of forgiveness may have different effects on forgiveness. The former may facilitate forgiveness, whereas the latter may inhibit forgiveness. The belief in unconditional forgiveness suggests that forgiveness is granted based on virtues such as generosity, hope, and moral love [31,46], and does not necessarily require an offender’s apology or repentance [21,22,47]. Thus, this belief can allow a victim to initiate the forgiveness process, regardless of whether the offender performs particular acts of contrition. In contrast, viewing apologies or amends from the offender as required conditions before forgiveness can be granted may leave the victim ensnared in bitterness and resentment because the offender may not perform the anticipated acts of contrition or may not perform them satisfactorily [23,24]. In addition, waiting for the offender to make amends means giving the offender more power to control the relationship [22]. This may decrease the victims’ sense of agency and increase their vigilance. Considering these two reasons, belief in conditional forgiveness may increase barriers to offering forgiveness. Supporting these claims, previous studies have demonstrated that belief in unconditional forgiveness is positively related to forgiveness, whereas belief in conditional forgiveness shows a lower correlation or is negatively related to forgiveness [21,22].

Forgiveness plays a central role in conflict management and the restoration of damaged interpersonal relationships [9,48,49]. It can foster constructive and cooperative acts (e.g., conciliation) between the parties involved in a broken relationship, thereby contributing to the maintenance of social relationships for future interactions [18,50,51,52]. Evidence has shown that forgiveness is negatively related to interpersonal avoidance [16]. It can help offended individuals overcome avoidance or revenge motivations toward a transgressor [17] and promote prosocial interactions. For example, previous research on close relationships has shown that, compared to individuals who have not forgiven an offender, individuals who have forgiven an offender tend to act on relational concerns, leading to more constructive communication with the offender [53]. In addition, individuals who have forgiven an offender are more likely to use the first-person plural pronouns (e.g., “us,” “we”) to describe their relationship with the offender, suggesting that forgiveness reduces psychological distance and enhances closeness with the offender [54].

Given that beliefs in unconditional and conditional forgiveness may facilitate and inhibit forgiveness, respectively, and that forgiveness reduces avoidance of the offender, we assume that:

**Hypothesis** **2a.** *Forgiveness plays a mediating role in the relationship between belief in unconditional forgiveness and avoidance of an offender*.

**Hypothesis** **2b.** *Forgiveness plays a mediating role in the relationship between belief in conditional forgiveness and avoidance of an offender*.

### 2.3. The Moderating Effect of Offender’s Explanation

Explanations can serve as a dispute resolution strategy [55]. When interpersonal offenses occur, offenders may offer explanations that typically fall into three categories: apology, excuse, and justification [56,57]. An apology is defined as the acknowledgement of responsibility for an interpersonal offense and the expression of remorse or regret toward the aggrieved victim [58,59]. An excuse is a self-serving explanation used by an offender to reduce their personal responsibility for an interpersonal offense [60]. This works by shifting the responsibility for the offense to an external and uncontrollable source [57]. Justification means that an offender takes full responsibility for an offense while claiming their act was appropriate because it aimed to accomplish superior moral goals [57]. For example, a university student offended a runner from another college before a race because the runner was found to be doping. The student admitted his responsibility for the offense but justified this by claiming that the runner’s behavior was unfair to the race.

Evidence has shown that explanations can be effective in restoring interpersonal relationships after offenses. For example, Shapiro (1991) [61] indicated that explanations (e.g., an excuse claiming a deception due to memory loss) can mitigate victims’ negative responses, such as unforgiveness and punitiveness, to deceptions. Research on close relationships showed that individuals who received an apology were more willing to forgive their partners than those who did not receive an apology [44,62]. Beyond close relationships, several studies have revealed that apologies from transgressors can reduce victims’ anger and aggression motivation while promoting forgiveness [28,59,63,64]. Furthermore, Mroz and Allen (2020) [56] found that, in transgression situations, people displayed more prosocial intentions (e.g., willingness to help in the future) toward transgressors who had provided an explanation (either an excuse or apology) compared with those who had not.

As elaborated in the previous two sections, people who hold a belief in unconditional forgiveness conceptualize that forgiveness can be granted without waiting for an explanation from the offender. Thus, their forgiveness and avoidance of the offender should not be influenced by whether or not the offender provides an explanation. In contrast, people who hold a belief in conditional forgiveness see the offender’s reparation and repentance as prerequisites before forgiveness can occur. Their forgiveness and avoidance of an offender would be influenced by the offender’s explanation. Specifically, when the victim does not receive an explanation from the offender, their belief in conditional forgiveness would decrease their willingness to forgive, and thus increase their avoidance of the offender. Conversely, when an aggrieved victim receives an explanation from the offender, their belief in conditional forgiveness would not influence their forgiveness or avoidance of the offender. Hence, we assume that:

**Hypothesis** **3a.** *The offender’s explanation does not moderate the relationship between belief in unconditional forgiveness and forgiveness and the relationship between belief in unconditional forgiveness and avoidance of an offender*.

**Hypothesis** **3b.** *The offender’s explanation plays a moderating role in the relationship between belief in conditional forgiveness and forgiveness and the relationship between belief in conditional forgiveness and avoidance of an offender*.

### 2.4. The Present Study

**Figure 1:**
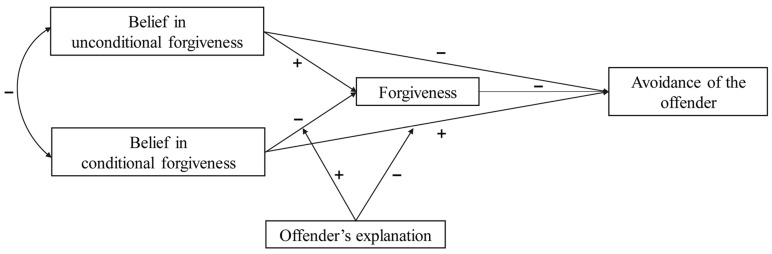
The hypothesized moderated mediation model.

This study aimed to investigate how college students’ beliefs regarding the nature of forgiveness (unconditional and conditional) influence their avoidance of an offender. We built a moderated mediation model to examine the mediating role of forgiveness and the moderating role of offender’s explanation in the relationships between beliefs in unconditional and conditional forgiveness and avoidance of an offender (see Figure 1).

## 3. Method

### 3.1. Participants and Procedures

A participant recruitment advertisement targeting college students was posted on two common Chinese social media platforms: Wechat and Weibo. College students voluntarily participated in the study by accessing an online survey provided in the advertisement. Before participants completed the online survey, they were required to carefully read and electronically sign an online informed consent form. They were given the option to withdraw from the study if they experienced psychological distress or chose not to participate at any point during the course of the study.

A total of 450 Chinese college students completed the survey using the Wenjuanxing online data collection platform. The survey asked participants to recall an unforgettable interpersonal offense. After this recollection, the offender’s explanation, participants’ beliefs about the unconditional and conditional nature of forgiveness, their forgiveness, and avoidance of the offender were assessed. Afterward, their social desirability and demographics were collected.

Twenty-seven participants (6%) were excluded because they did not answer carefully; for example, they failed preplanned attention checks (e.g., “Please select response 1 for this item”) or gave the same answer for all items. Of the remaining 423 participants, 57.9% were females and 42.1% were males. Their average age was 20.90 years (*SD* = 1.88, range: 18–26).

### 3.2. Measures

The English version of the scales used in this study was translated using standard translation and back-translation procedures.

#### 3.2.1. Interpersonal Offense

Participants recalled an unforgettable incident in which they had been offended by another person (e.g., classmate, friend, teacher, or family member). They were then asked to answer the following items: 1. The time since the offense (a single item asking how many months had passed since the offense); 2. the frequency of the offense (1 = *this was the first time*, 2 = *it happened on occasions*, 3 = *it was frequent*, 4 = *it is on-going*); 3. the offense’s severity (1 = *not serious at all* to 5 = *extremely serious*); 4. perceived intention of the offender (1 = *no intention to offend* to 5 = *full intention to offend*); 5. offender’s power (1 = *low power offender*, 2 = *peer*, 3 = *high power offender*). As these five factors have been found to influence victims’ forgiveness and behavioral reactions in previous research [16,21], they were measured as control variables in this study.

#### 3.2.2. Offender’s Explanation

The offender’s explanation was assessed using a dichotomous question. The participants were asked to indicate whether the offender had provided an explanation regarding the offense (0 = *no explanation*, 1 = *provided an explanation*).

#### 3.2.3. Beliefs about the Unconditional and Conditional Nature of Forgiveness

The two-dimensional Conditional–Unconditional Forgiveness Scale [22] was used. The scale includes eight items in two subscales − unconditional and conditional, with four items each. An example item measuring belief in unconditional forgiveness is “A person does not have to change for the better before I can forgive them,” while an example item measuring belief in conditional forgiveness is “Before I can forgive someone for an offense, they have to repent in some way”. The items were rated on five-point scales (1 = *strongly disagree* to 5 = *strongly agree*), with higher scores representing a stronger belief in unconditional and conditional forgiveness, respectively. In this study, the Cronbach’s α for the beliefs in unconditional and conditional forgiveness were 0.88 and 0.86, respectively.

#### 3.2.4. Forgiveness

The Chinese version of the Forgiveness Scale [65] was used to measure participants’ forgiveness of the interpersonal offense that they had recalled. The scale contains 13 items. An example item is “I have been able to let go of my anger toward the person who wronged me”. Each item was rated on a five-point scale (1 = *strongly disagree* to 5 = *strongly agree*), with higher scores representing a higher level of forgiveness. In this study, the Cronbach’s α for the scale was 0.92.

#### 3.2.5. Avoidance of the Offender

The subscale of avoidance from the Transgression-related Interpersonal Motivations Inventory [9] was adapted to measure participants’ avoidance of the offender. An example item is “I keep as much distance between the offender and me as possible”. The subscale includes seven items, which were rated on five-point scales (1 = *strongly disagree* to 5 = *strongly agree*). Higher scores represent greater avoidance of offenders. The Cronbach’s α for the scale was 0.90 in this study.

#### 3.2.6. Social Desirability and Demographics

Social desirability was assessed as a control variable. The 13-item short form (Form C) of the Marlowe–Crown Scale [66] was used. An example item is “No matter who I am talking to, I am always a good listener”. Participants rated each item as either 0 = *false* or 1 = *true*. Participants’ gender (1 = *male*, 2 = *female*) and age were measured.

### 3.3. Data-Analytical Approach

First, a common method bias test was performed using SPSS 25.0 to examine whether the current study suffered from a common method bias issue. Second, a confirmatory factor analysis (CFA) was conducted in Mplus 7.4 to test the validity of the four study variables, including belief in unconditional forgiveness, belief in conditional forgiveness, forgiveness, and avoidance of the offender. Descriptive statistics and Pearson’s correlation analyses were then performed using SPSS 25.0.

Furthermore, path analyses were conducted in Mplus 7.4 to test the hypotheses. Specifically, we first tested whether the two predictors (belief in unconditional forgiveness and belief in conditional forgiveness) affected the outcome variable (avoidance of the offender; Hypotheses 1a and 1b). Second, we examined whether the two predictors indirectly affected the outcome variable through the mediator (forgiveness) (mediation test for Hypotheses 2a and 2b). Third, we assessed whether the moderator (offender’s explanation) moderated the effects of the two predictors on the mediator and the effects of the two predictors on the outcome variable (moderated mediation test for Hypotheses 3a and 3b).

## 4. Results

### 4.1. Common Method Bias Testing

As the research data were collected using a self-reported questionnaire, there was the possibility of a common method bias issue. The Harman single factor test [67] showed a KMO value of 0.91 (*p* < 0.001), indicating that the data were suitable for factor analysis. There were five factors with eigenvalues greater than 1, and the first factor showed a variance of 16.20%, which did not reach the criterion of 40%. Thus, this study did not have a serious common method bias problem.

### 4.2. Discrimination Testing

The CFA revealed that the four-factor model (belief in unconditional forgiveness, belief in conditional forgiveness, forgiveness, and avoidance of the offender) had a good fit (*χ*^2^(344) = 949.30, *χ*^2^/df = 2.76, CFI = 0.91, TLI = 0.90, RMSEA = 0.06, SRMR = 0.05). Furthermore, as shown in Table 1, the four-factor model displayed a better fit than the alternative models. These results indicated that belief in unconditional forgiveness, belief in conditional forgiveness, forgiveness, and avoidance of the offender showed sufficient discriminatory ability in this study.

### 4.3. Descriptive Statistics

Table 2 presents the results of the descriptive statistics and correlation analyses. As expected, beliefs in unconditional and conditional forgiveness were negatively correlated (*r* = −0.15, *p* = 0.003). Belief in unconditional forgiveness was positively related to forgiveness (*r* = 0.41, *p* < 0.001) and negatively related to avoidance of the offender (*r* = −0.20, *p* < 0.001). Belief in conditional forgiveness was negatively related to forgiveness (*r* = −0.23, *p* < 0.001) and positively related to avoidance of the offender (*r* = 0.27, *p* < 0.001). Forgiveness was negatively related to avoidance of the offender (*r* = −0.38, *p* < 0.001).

### 4.4. Hypothesis Testing

Data were analyzed using the bias-corrected percentile bootstrap method (1000 samples) in Mplus 7.4. First, the effects of beliefs in unconditional and conditional forgiveness on avoidance of the offender were examined. The results (Table 3, M_1_) showed that, after controlling for gender, age, five offense-related variables, and social desirability, participants’ belief in unconditional forgiveness negatively predicted their avoidance of the offender (*β* = −0.17, *SE* = 0.06, *t* = −3.06, *p* = 0.002, 95% CI = −0.27 to −0.06). In contrast, belief in conditional forgiveness positively predicted avoidance of the offender (*β* = 0.23, *SE* = 0.06, *t* = 3.75, *p* < 0.001, 95% CI = 0.11 to 0.34). Thus, Hypotheses 1a and 1b are supported.

Second, the mediating role of forgiveness was examined. As shown in Table 3, when the meditator variable (forgiveness) was added to the model as a predictor (M_3_), it negatively predicted avoidance of the offender (*β* = −0.34, *SE* = 0.06, *t* = −5.35, *p* < 0.001, 95% CI = −0.46 to −0.21), and the direct predictive effect of belief in unconditional forgiveness on avoidance of the offender became non-significant (*β* = −0.04, *SE* = 0.06, *t* = −0.65, *p* = 0.513, 95% CI = −0.16 to 0.08). The direct predictive effect of belief in conditional forgiveness remained significant (*β* = 0.16, *SE* = 0.06, *t* = 2.70, *p* = 0.007, 95% CI = 0.05 to 0.28) when the mediator variable was included in the model. The indirect effect of belief in unconditional forgiveness on avoidance of the offender via forgiveness was −0.13 (*SE* = 0.03, 95% CI = −0.19 to −0.08), accounting for 76.5% of the total effect of the belief in unconditional forgiveness. The indirect effect of belief in conditional forgiveness on avoidance of the offender via forgiveness was 0.07 (*SE* = 0.02, 95% CI = 0.04 to 0.12), accounting for 30.4% of the total effect of the belief in conditional forgiveness. Hence, Hypotheses 2a and 2b are supported.

**Figure 2:**
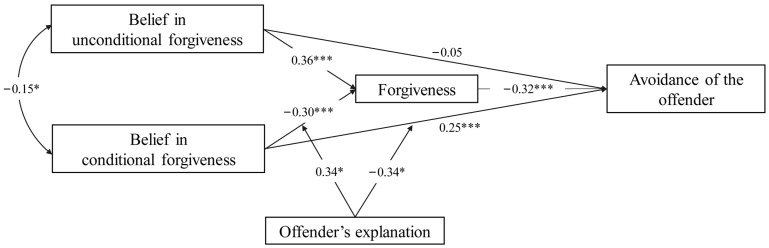
Indirect pathway coefficients for the relationships between beliefs about the nature of forgiveness and avoidance of the offender via forgiveness, conditional on offender’s explanation. * *p* < 0.05, *** *p* < 0.001.

Third, a moderated mediation model with the offender’s explanation as the moderator was tested. The results demonstrated that, after the offender’s explanation was added to the model, there was no statistically significant interaction between belief in unconditional forgiveness and explanation on forgiveness (*β* = −0.02, *SE* = 0.15, *t* = −0.16, *p* = 0.873, 95% CI = −0.30 to 0.29; Table 4 M_4_). The interaction between belief in unconditional forgiveness and explanation on avoidance of the offender was also not significant (*β* = 0.07, *SE* = 0.17, *t* = 0.43, *p* = 0.664, 95% CI = −0.27 to 0.40; Table 4 M_5_). In contrast, there was a significant interaction between belief in conditional forgiveness and explanation on forgiveness (*β* = 0.34, *SE* = 0.15, *t* = 2.34, *p* = 0.019, 95% CI = 0.04 to 0.62; Table 4 M_4_ and Figure 2). In addition, the interaction between belief in conditional forgiveness and explanation on avoidance of the offender was significant (*β* = −0.34, *SE* = 0.16, *t* = −2.12, *p* = 0.034, 95% CI = −0.66 to −0.01; Table 4 M_5_ and Figure 2). This suggests that the offender’s explanation moderates the association between belief in conditional (but not unconditional) forgiveness and forgiveness, as well as avoidance of the offender. Therefore, Hypotheses 3a and 3b are supported.

Furthermore, simple slope analyses indicated that when participants had not received an explanation from the offender, their belief in conditional forgiveness negatively predicted forgiveness (*β* = −0.30, *SE* = 0.06, *t* = −5.27, *p* < 0.001, 95% CI = −0.41 to −0.18). However, when they received an explanation from the offender, their belief in conditional forgiveness did not have a significant predictive effect on forgiveness (*β* = −0.10, *SE* = 0.08, *t* = −1.35, *p* = 0.176, 95% CI = −0.24 to 0.06). Regarding avoidance of the offender, when participants had not received an explanation from the offender, their belief in conditional forgiveness positively predicted their avoidance of the offender (*β* = 0.25, *SE* = 0.07, *t* = 3.74, *p* < 0.001, 95% CI = 0.13 to 0.40). However, when they received an explanation from the offender, their belief in conditional forgiveness did not significantly influence their avoidance of the offender (*β* = 0.05, *SE* = 0.08, *t* = 0.63, *p* = 0.527, 95% CI = −0.10 to 0.22). In addition, the mediating effect of forgiveness in the relationship between the belief in conditional forgiveness and the avoidance of the offender differed under the conditions of having and not having received an explanation (Table 5). These results suggest that an explanation from the offender can reduce the negative effect of the belief in conditional forgiveness on forgiveness, which thus alleviates behavioral avoidance of the offender.

## 5. Discussion

This study aimed to investigate the effect of individuals’ beliefs about the nature of forgiveness (unconditional and conditional) on their avoidance of interpersonal offenses, as well as the mediating role of forgiveness and the moderating role of whether the offender provides an explanation. Considering that previous research did not adequately examine the effect of beliefs in unconditional and conditional forgiveness among collectivistic non-Western cultures, this study was conducted within the context of China, a representative collectivistic country. Furthermore, given the prevalence of interpersonal conflicts in college life [28,29], and the tendency for avoidance as a typical behavioral response to offenses during early adulthood [6,30], this study focused on Chinese college students as participants. As hypothesized, college students’ belief in unconditional forgiveness negatively predicts their tendency to avoid an offender, whereas their belief in conditional forgiveness positively predicts avoidance of an offender. Additionally, beliefs in unconditional and conditional forgiveness influence avoidance of an offender through the mediating factor of forgiveness. Individuals with a strong belief in unconditional forgiveness decrease their avoidance of an offender by fostering forgiveness, whereas those with a strong belief in conditional forgiveness increase their avoidance of an offender by hindering forgiveness. Moreover, when the offender provides an explanation, such as an apology, excuse, or justification, individuals with a strong belief in conditional forgiveness are more likely to grant forgiveness, leading to a decrease in avoidance tendencies toward the offender.

### 5.1. The Divergent Effects of Beliefs about the Nature of Forgiveness on Avoidance of an Offender

This study found divergent effects for beliefs about the nature of forgiveness on avoidance of an offender. The belief that forgiveness is unconditional decreases avoidance of an offender, whereas the belief that forgiveness is conditional (e.g., requiring the offender to perform acts of contrition before forgiveness can be offered) increases the avoidance of an offender. These findings are consistent with previous research on beliefs about the nature of forgiveness. Krause and Ellison (2003) [23] showed that individuals who demand an act of remorse from their transgressors experience more psychological stress than those who forgive unconditionally. They suggested that the belief in unconditional forgiveness can help victims more quickly let go of negative emotions and ruminations related to interpersonal offenses. Toussaint et al. (2012) [24] demonstrated that the belief in conditional (vs. unconditional) forgiveness of others predicts a higher risk of death due to its adverse impacts on physical health. Gismero-González et al. (2020) [21] found that belief in unconditional forgiveness promotes psychological well-being, whereas belief in conditional forgiveness decreases it. Although these studies only showed the effects of beliefs in unconditional and conditional forgiveness on mental health, we believe our findings are in line with this, given that good mental health (e.g., high optimism and resilience, and low depression) can promote pro-relationship responses to interpersonal offenses [19,68,69].

### 5.2. The Mediating Role of Forgiveness

This study revealed that beliefs in unconditional and conditional forgiveness influence avoidance of an offender through the mediator of forgiveness. According to the stress-and-coping model of forgiveness [70], a stressful event (such as an interpersonal offense) induces emotional reactions (e.g., fear and anger) that can further influence individuals’ behaviors (e.g., interpersonal avoidance) as they try to cope with the stress. Forgiveness is an intrapersonal process of emotional regulation (e.g., reducing negative emotions or increasing positive emotions toward a wrongdoer) that aims to reduce stress [15,71]. Therefore, forgiveness can play a mediating role in the link between a stressful offense and an individual’s behavioral response to the offense. The stress-and-coping model of forgiveness proposes that individual characteristics influence the coping process [70]. Our findings are consistent with the model showing that belief in unconditional forgiveness decreases the avoidance of an offender by promoting forgiveness, whereas belief in conditional forgiveness increases the avoidance of an offender by impeding forgiveness. These two types of forgiveness belief can impact forgiveness for the following reasons: as belief in unconditional forgiveness means that forgiveness can be granted unconditionally, it allows a victim to initiate the forgiveness process autonomously, independently of the offender’s behavior. In contrast, belief in conditional forgiveness requires the offender to perform acts of repentance, which may inhibit the initiation of forgiveness because the offender may not fulfill the prerequisites of forgiveness (e.g., the offender may not be aware of their hurtful act and may not make any amends). Moreover, requiring the offender to perform acts of repentance means shifting more power to them to control the situation, which may decrease victims’ sense of agency and intensify the constraints and stress they feel. In this situation, positive emotional regulation (e.g., forgiveness) does not occur easily. The divergent processes of emotional regulation driven by beliefs in unconditional and conditional forgiveness ultimately translate into behavioral reactions, such as the extent to which victims avoid the offender.

### 5.3. The Moderating Role of Offender’s Explanation

This study found that an offender’s explanation moderates the relationship between belief in conditional forgiveness and forgiveness, as well as the relationship between belief in conditional forgiveness and avoidance of an offender. Belief in conditional forgiveness requires the offender to take responsibility for their actions and make amends before forgiveness can be granted. This puts the offender in a position of control over the situation, potentially decreasing the victim’s sense of agency and exacerbating negative emotions and stress following the offense. An offender’s explanation can demonstrate that the offender is taking responsibility for their behavior, which can increase the victim’s sense of agency and control over the situation, leading to more positive emotions, greater willingness to forgive, and less avoidance of the offender. In addition, belief in conditional forgiveness may increase the victim’s expectation that the offender will provide a satisfactory explanation or apology. If the offender provides a convincing explanation, the victim can make better sense of the offense and will be more likely to forgive and reduce their avoidance of the offender. Conversely, if the offender fails to provide a satisfactory explanation or apology, the victim may perceive the offender as being insincere or not remorseful, leading to unforgiveness and increased avoidance of the offender. Therefore, the offender’s explanation can determine whether the victim will grant forgiveness and reduce avoidance.

We did not find evidence that the offender’s explanation moderated the relationship between belief in unconditional forgiveness and forgiveness, or between belief in unconditional forgiveness and avoidance of an offender. Belief in unconditional forgiveness emphasizes forgiveness as an intrapersonal process initiated by the victim, independent of the offender’s behavior or external conditions. Hence, the victim’s decision to forgive or avoid the offender is not influenced by the offender’s explanation. Consequently, the effects of belief in unconditional forgiveness on forgiveness and avoidance of the offender are not contingent on the offender’s explanation.

### 5.4. Implications and Contributions

First, this study provides initial evidence that individuals’ beliefs about the nature of forgiveness predicts how they respond to offenses and interact with offenders. Specifically, we found that belief in the unconditional nature of forgiveness is associated with decreased avoidance of the offender, whereas belief in the conditional nature of forgiveness is associated with increased avoidance of the offender. Previous research on beliefs about the nature of forgiveness has mostly focused on the benefits of belief in unconditional (vs. conditional) forgiveness for health and well-being [21,23,24]. This study enriches the literature by demonstrating that belief in unconditional (vs. conditional) forgiveness can also be effective in reducing negative responses and has important implications for the restoration of damaged interpersonal relationships.

Second, previous research showed that personal characteristics influence the extent to which people forgive an offense [20,72]. However, it is surprising that individuals’ beliefs about the nature of forgiveness did not receive sufficient attention in the literature [22]. It remains unknown whether these beliefs affect collectivistic non-Western cultures, where forgiveness is influenced not only by personal characteristics but also by cultural expectations of maintaining social harmony [25,26,27]. This study initially demonstrated that personal beliefs about the nature of forgiveness influence forgiveness in collectivistic cultures. This emphasizes the importance of considering individual differences in beliefs about forgiveness when promoting forgiveness. For individuals who believe in unconditional forgiveness, promoting forgiveness may involve encouraging them to focus on their own internal processes of forgiveness rather than on the offender’s behavior. This could include promoting self-reflection, acceptance of the offense, and the decision to move on from the hurt caused by the offense, which are referred to as intrapersonal interventions. Conversely, for those who believe in conditional forgiveness, promoting forgiveness may require facilitating communication between the victim and offender, ensuring that the offender takes responsibility for their actions and makes amends. These are referred to as interpersonal interventions. By doing this, a better understanding can be fostered between the involved parties, which can potentially increase the victim’s willingness to forgive.

Third, this study highlights the importance of the offender’s explanation in moderating the effects of belief in conditional forgiveness on forgiveness and avoidance of the offender. Although belief in conditional forgiveness may erect barriers to the forgiveness process and result in avoidance of the offender, we found a condition where the negative impacts of belief in conditional forgiveness can be alleviated. An offender’s explanation can promote forgiveness and reduce avoidance among individuals with a strong belief in conditional forgiveness. This finding offers insights into the possibility of developing effective interventions, such as creating opportunities for victims and offenders to communicate with each other regarding interpersonal offenses. Such interventions can help individuals who believe in conditional forgiveness let go of negative emotions and ruminations connected to offenses, thereby resolving interpersonal conflicts and promoting positive relationships.

### 5.5. Limitations and Future Directions

This study has several limitations that require further investigation.

First, our conceptualizations of beliefs in unconditional and conditional forgiveness were based on previous studies that emphasized that the core difference between unconditional and conditional forgiveness lies in the presence or absence of an exchange-like process [21,22,23,24,47]. Conditional forgiveness is contingent upon certain actions or changes from the offender. It entails a bilateral relationship between the forgiver and offender based on the condition that the offender acknowledges their wrongdoing, apologizes, and repents. Conversely, unconditional forgiveness transcends the boundaries of what can be forgiven and is granted *without any conditions or requirements*. However, according to these conceptualizations, it may seem that unconditional forgiveness is implausible in the public and social realm [73]. Even if it does occur, it could be risky and potentially have negative consequences, such as repeated offenses in the future and threats to victims’ dignity and moral value [23,74,75]. Consequently, scholars have been exploring how to implement unconditional forgiveness in a more realistic manner. One approach to making unconditional forgiveness tangible in the social realm is to reflect on the underlying virtues associated with it: modesty, magnanimity, compassion, and humility. These virtues imply acting for the development, improvement, and service of others [76]. In this view of unconditional forgiveness, forgivers move beyond resentment, exhibiting concerns for the well-being and personal growth of others, including the offender [77]. Giannini (2017) proposes that forgiveness should be grounded in hope [46]. According to Giannini, unconditional forgiveness is driven by a genuine belief in the offender’s capacity for positive change and desire for such change. The forgiver chooses to let go of negative emotions and resentment, extending forgiveness with the hope for the offender’s transformation. Moreover, within the framework of unconditional forgiveness grounded in hope, forgivers maintain a balanced reliance on the hoped-for outcomes. This means that, while hoping for the offender’s positive change, forgivers remain realistic and prepared for the possibilities that the change may not occur or there may be obstacles along the way [46]. This perspective can help explain why forgiveness is sometimes granted, but its expression depends on the attitude of the offender. The intention behind this approach is to encourage the offender to mature and repent. In this sense, unconditional forgiveness grounded in hope balances the forgiver’s compassionate concern for the offender with an awareness of the uncertainties and risks involved in forgiveness. In this study, we solely focused on beliefs about forgiveness *without any conditions or requirements*. However, we inadvertently overlooked belief in unconditional forgiveness, which involves kindness toward offenders and aims to promote positive change and personal growth. Future research should delve deeper into this aspect and examine it more comprehensively.

Second, this study only focused on Chinese college students. Owing to cultural contexts and age, individuals’ beliefs about the nature of forgiveness, the explanatory strategies they adopt, and their forgiveness and interpersonal reactions in response to offenses may vary. For example, some studies have found that younger individuals are more likely to avoid an offender than older individuals [6], while other research has arrived at the opposite conclusion [78]. Future studies should recruit participants from diverse cultural backgrounds and expand the study to a wider age range.

Third, the present study demonstrated that forgiveness mediates the relationship between beliefs in unconditional and conditional forgiveness and avoidance of the offender. Forgiveness comprises two distinct processes that regulate negative and positive emotions. The forgiveness process for negative emotions involves overcoming negative emotions (e.g., anger and hatred) toward the wrongdoer, whereas the forgiveness process for positive emotions involves increasing positive emotions (e.g., compassion and empathy) toward the wrongdoer [15,65]. Future research could delve deeper into investigating whether beliefs in unconditional and conditional forgiveness affect behavioral responses to interpersonal offenses through the forgiveness process of regulating negative, positive, or both emotions.

In addition, the current study initially revealed that an explanation provided by the offender can ameliorate the negative effects of the belief in conditional forgiveness on the victim’s forgiveness and interactions with the offender. Various types of explanations, including apologies, excuses, and justifications, have been identified in the literature [56,57]. Researchers have argued that different types of offender explanations may have varying effects on the victim’s affective and behavioral reactions to an offense, according to the attribution theory [56]. For example, an apology where the offender takes full responsibility for the offense may not have a significant effect on reducing the victim’s negative reactions, but may facilitate more positive reactions. Conversely, excuses in which the offender shifts the responsibility for the offense to external and uncontrollable sources may reduce the victim’s negative reactions [79]. Therefore, further empirical research is needed to distinguish the moderating effects of the type of explanation on the relationships between belief in conditional forgiveness and forgiveness, as well as interactions with the offender. This line of investigation will offer specific suggestions for resolving interpersonal conflicts.

## 6. Conclusions

Previous research on beliefs about the nature of forgiveness has primarily shown that beliefs in unconditional and conditional forgiveness have distinct effects on health and well-being. However, little is known about how they influence victims’ responses to interpersonal offenses and their interactions with offenders. In this study, we recruited Chinese college students as participants and demonstrated that: (1) a victim’s belief in unconditional forgiveness negatively predicts their avoidance of an offender, whereas their belief in conditional forgiveness positively predicts the avoidance of an offender; (2) forgiveness mediates the relationships between beliefs in unconditional and conditional forgiveness and avoidance of an offender; (3) the offender’s explanation moderates the relationships between belief in conditional forgiveness and forgiveness, as well as avoidance of an offender. These findings suggest that belief in unconditional forgiveness can benefit victims by reducing their negative reactions to interpersonal offenses, which may help to repair damaged interpersonal relationships. They also highlight the role of the offender’s explanation in reducing avoidance, and therefore, promoting conflict resolution among victims who believe in conditional forgiveness.

## Figures and Tables

**Table 1:** Results of confirmatory factor analysis.

Models	*χ* ^2^	df	*χ*^2^/df	CFI	TLI	RMSEA	SRMR
4-factor model (BUF, BCF, F, A)	949.30	344	2.76	0.91	0.90	0.06	0.05
3-factor model (BUF + F, BCF, A)	1638.96	347	4.72	0.80	0.78	0.09	0.08
3-factor model (BCF + F, BUF, A)	1666.24	347	4.80	0.79	0.78	0.10	0.09
3-factor model (BUF + BCF, F, A)	1707.68	347	4.92	0.79	0.77	0.10	0.10
2-factor model (BUF + BCF + F, A)	2353.72	349	6.74	0.69	0.66	0.12	0.10
2-factor model (BUF + BCF, F + A)	2917.13	349	8.36	0.60	0.57	0.13	0.13
1-factor model (BUF + BCF + F + A)	3546.44	350	10.13	0.50	0.46	0.15	0.14

Note. BUF = belief in unconditional forgiveness; BCF = belief in conditional forgiveness; F = forgiveness; A = avoidance of the offender.

**Table 2:** Means, standard deviations, and inter-correlations among study variables (*N* = 423).

Variable	1	2	3	4	5	6	7	8	9	10	11	12	13
1. Unconditional ^a^	1.00												
2. Conditional ^b^	−0.15 **	1.00											
3. Forgiveness	0.41 ***	−0.23 ***	1.00										
4. Explanation ^c^	−0.01	0.01	0.09	1.00									
5. Avoidance	−0.20 ***	0.27 ***	−0.38 ***	−0.02	1.00								
6. Gender ^d^	0.18 ***	−0.04	−0.02	−0.01	−0.04	1.00							
7. Age	0.05	−0.05	0.03	−0.08	−0.04	0.02	1.00						
8. Time	0.02	−0.05	0.03	0.03	−0.07	−0.04	−0.06	1.00					
9. Frequency	−0.01	0.03	−0.06	−0.19 ***	0.01	0.10 *	−0.12 *	−0.10 *	1.00				
10. Severity	−0.04	−0.21 ***	−0.13 **	0.01	−0.08	0.04	0.01	0.17 ***	0.05	1.00			
11. Intention	−0.02	−0.24 ***	−0.04	−0.06	−0.12 *	0.06	0.05	0.03	0.02	0.55 ***	1.00		
12. Offender’s power ^e^	−0.14 **	0.05	−0.07	0.03	0.02	−0.13 **	0.03	−0.08	0.04	−0.01	−0.06	1.00	
13. Social desirability	−0.00	−0.05	−0.06	−0.08	−0.08	0.13 **	0.01	0.00	0.13 *	0.02	0.04	0.06	1.00
*M*	3.24	2.39	45.82	0.54	16.71	1.58	20.90	3.97	1.66	3.39	3.23	2.01	7.62
*SD*	1.01	0.80	9.60	0.50	5.78	0.49	1.88	4.08	0.74	1.13	1.19	0.59	2.39

Note. * *p* < 0.05, ** *p* < 0.01, *** *p* < 0.001. ^a^ Belief in unconditional forgiveness. ^b^ Belief in conditional forgiveness. ^c^ Explanation: 0 = no explanation, 1 = offender provided an explanation. ^d^ Gender: 1 = male, 2 = female. ^e^ Offender’s status: 1 = low-power offender, 2 = peer, 3 = high-power offender.

**Table 3:** Mediation effect test of forgiveness (*N* = 423).

Variable	Avoidance (M_1_)		Forgiveness (M_2_)		Avoidance (M_3_)	
	*β*	*SE*	*β*	*SE*	*β*	*SE*
Gender	0.01	0.05	−0.08	0.05	−0.02	0.05
Age	−0.02	0.05	0.01	0.05	−0.01	0.04
Time	−0.06	0.04	0.04	0.04	−0.05	0.04
Frequency	0.00	0.06	−0.02	0.06	−0.01	0.05
Severity	0.01	0.06	−0.17 **	0.06	−0.05	0.06
Intention	−0.07	0.07	0.02	0.06	−0.07	0.06
Offender’s power	−0.02	0.05	−0.01	0.05	−0.02	0.05
Social desirability	−0.06	0.05	−0.06	0.05	−0.08	0.04
Unconditional	−0.17 **	0.06	0.38 ***	0.04	−0.04	0.06
Conditional	0.23 ***	0.06	−0.21 ***	0.05	0.16 **	0.06
Forgiveness					−0.34 ***	0.06
*R* ^2^	0.10 **		0.25 ***		0.19 ***	

Note. ** *p* < 0.01, *** *p* < 0.001.

**Table 4:** Moderation effect test of explanation (*N* = 423).

Variable	Forgiveness (M_4_)		Avoidance (M_5_)	
	*β*	*SE*	*β*	*SE*
Gender	−0.07	0.04	−0.02	0.05
Age	0.02	0.05	−0.01	0.04
Time	0.04	0.04	−0.05	0.04
Frequency	0.00	0.05	−0.01	0.05
Severity	−0.18 **	0.05	−0.03	0.06
Intention	0.04	0.06	−0.07	0.06
Offender’s power	−0.01	0.05	−0.02	0.04
Social desirability	−0.05	0.04	−0.07	0.04
Unconditional	0.36 ***	0.06	−0.05	0.08
Conditional	−0.30 ***	0.06	0.25 ***	0.07
Explanation	−0.20	0.18	0.23	0.24
Unconditional × Explanation	−0.02	0.15	0.07	0.17
Conditional × Explanation	0.34 *	0.15	−0.34 *	0.16
Forgiveness			−0.32 ***	0.06
*R* ^2^	0.32 ***		0.28 ***	

Note. * *p* < 0.05, ** *p* < 0.01, *** *p* < 0.001.

**Table 5:** Conditional indirect effects of the belief of conditional forgiveness on avoidance.

Outcome	Condition	Indirect Effects of Conditional Forgiveness Belief via Forgiveness		
		Estimate	*SE*	95% CI
Avoidance	Did not receive an explanation	0.73	0.21	[0.41, 1.27]
	Received an explanation	0.23	0.17	[−0.11, 0.58]
	Difference	−0.50	0.25	[−1.11, −0.11]

## Data Availability

The data and materials of the present study supporting the conclusions are available from the corresponding author on request.
